# Vibrational strong coupling modulated by graphene plasmons in deep metal grating structures

**DOI:** 10.1515/nanoph-2025-0275

**Published:** 2025-09-15

**Authors:** Md Faysal Hossain, Wonmi Ahn

**Affiliations:** UNAM – National Nanotechnology Research Center and Institute of Materials Science and Nanotechnology, 52948 Bilkent University, Ankara, Türkiye

**Keywords:** vibrational strong coupling, cavity polaritons, vacuum Rabi splitting, deep metal grating, magnetic polaritons, graphene plasmons

## Abstract

We present a novel design approach for vibrational strong coupling (VSC) that enables spectrally accessible and controllable vibrational-polaritonic states using a graphene-integrated deep silver (Ag) grating. The deep Ag grating supports strong infrared resonances arising from hybrid magnetic polariton and surface plasmon modes, facilitating coherent coupling with the molecular vibrations of a test molecule, poly(methyl methacrylate) (PMMA). Integrating graphene into the deep Ag grating introduces discrete graphene plasmon (GP) modes that interact with the vibrational-polaritonic modes, providing spectral tunability and control over otherwise static polaritonic states. Consequently, the upper and lower polaritonic modes split into two distinct branches due to the sharp GP modes. The mixing ratio among the grating mode, molecular vibration, and GP mode is significantly modulated by adjusting the chemical potential applied to the graphene and varying the number of graphene layers incorporated into the grating. This ability to spectrally access and control polaritonic states makes the graphene-integrated Ag grating a promising platform for VSC applications, which potentially enables the use of polaritonic states as distinct quantum states for polaritonic chemistry.

## 1 Introduction

Strong coupling between photons and material transitions inside an optical cavity offers novel ways to modify material properties [1], [2]. The most widely studied example is the strong coupling of cavity photons with excitonic materials such as *j*-aggregates [3], quantum dots [4], [5], and organic dye molecules [6]. When optical fields and excitonic transitions exchange energies faster than their respective decay rates, new hybrid light–matter energy states – called polaritonic states – are formed [7]. It has been demonstrated that the excitonic–polaritonic states influence nonlinear optical phenomena such as Bose–Einstein condensation [8], low-threshold lasing [9], and superfluidity [10], opening new possibilities for polaritonic quantum processing. However, strong coupling is not limited to excitonic materials; it also extends to materials exhibiting strong vibrational transitions [11], [12]. Through vibrational strong coupling (VSC), the rates [13], [14] and products [15] of chemical reactions are altered, demonstrating the potential to synthesize chemical compounds with fewer by-products and to create new reaction pathways [16], [17], [18].

Polaritons are bosonic quasiparticles that exhibit properties of both light and matter. Their angular dispersion [19] and delocalized nature [20] arise from the photonic characteristics of the polariton. At the same time, polaritons inherit properties of material excitations, enabling them to influence and modify potential energy surfaces [21] and chemical reaction pathways [14], [15], as well as interact with other material excitations [22]. The varying photonic and material contributions of polaritons are reflected in the upper and lower polaritonic (UP and LP) modes observed in the cavity dispersion and are quantitatively described by the Hopfield coefficients [23]. Standard Fabry–Pérot (FP) microcavities have been widely used to generate dispersing UP and LP modes under varied angle of incident light, producing polaritons with more light-like or material-like character relative to the original material resonance [24], [25]. Plasmonic cavity structures have also been used to confine polaritonic states within nanoscale volumes, pushing the limits of strong coupling down to the single-molecule level [26].

Going beyond the mere generation, it is critical to spectrally access and control polaritonic states, as this capability could enable the use of polaritonic states as distinct quantum states. However, conventional FP and plasmonic cavities do not offer spectral access to individual polaritonic modes; instead, they simply produce broad cavity resonances that split into UP and LP modes under strong coupling. To spectrally access or manipulate specific UP and LP modes, the coupled system requires additional discrete resonance modes that can be independently tuned to couple with the polaritonic states. Introducing a second resonance mode into the coupled system aligns with the concept of multimode strong coupling [27], [28], [29], [30]. However, most studies on multimode strong coupling focus primarily on demonstrating strong interactions between multiple oscillators rather than on spectral control. Achieving spectral access and control over hybrid light–matter polaritonic states could provide many new perspectives for understanding fundamental properties of the polaritonic states and expanding their applications. For example, it is expected to enable detailed studies of relaxation pathways in strongly coupled organic molecules at specific in-plane wavevectors (*k*_‖_) [31], reveal exciton transport distances as a function of their polaritonic versus excitonic character [32], and clarify how vibrational-polaritonic states influence reaction kinetics at defined *k*_‖_ [33].

In this study, we used a deep metal grating structure integrated with graphene as an infrared optical resonator to access and manipulate specific polaritonic modes. While shallow metal gratings with depths ranging from 30 to 100 nm have previously been used for VSC [34], [35], [36], our metal grating features a significantly greater depth, ranging from 0.5 to 1.1 μm. In such deep metal gratings, magnetic resonances arise from antiparallel current loops induced by the magnetic field of the incident wave, producing magnetic polaritons (MPs) [37], [38]. In addition to the MP modes, the deep metal grating supports surface plasmon (SP) modes, resulting in high-*Q* infrared optical modes that enable VSC with a molecular absorber of our choice, poly(methyl methacrylate) (PMMA). The grating modes, arising from the hybridization of MP and SP resonances, can be tuned by adjusting structural parameters such as the period, trench gap, grating depth, and the materials used to fabricate the grating. In earlier studies, deep metal gratings have been used to enhance thermophotovoltaic power efficiency [39] and near-infrared light absorption in graphene [40], [41]. However, they have not been exploited to demonstrate VSC with molecular vibrations, despite their ability to generate spectrally and spatially tunable infrared resonances.

A unique feature of our design is the integration of graphene into the deep metal grating. The planar graphene layer placed over the grating generates much sharper graphene plasmon (GP) modes compared to graphene patterned into ribbons [42], [43], [44]. These discrete GP modes enable spectral access to polaritonic states, which can be tuned by adjusting the chemical potential applied to graphene or by varying the number of graphene layers. Consequently, the graphene-integrated deep metal grating allows dynamic control of vibrational-polaritonic states formed between grating modes and molecular vibrations. Additionally, graphene enhances coupling strength by positioning its layers at regions of maximum optical field intensity within the grating. Strong localized electric (*E*)-fields at the trench openings excite highly confined GP modes, further amplifying the coupling strength between grating modes and molecular vibrations. Finally, the mode volume (*V*_*m*_) is significantly reduced in the graphene-integrated deep metal grating, enabling the formation of vibrational-polaritonic states with only a few molecules located on or near the graphene layer.

Gaining spectral control over vibrational-polaritonic states holds significant potential for selectively modulating molecular properties in the strong coupling regime. In this study, we demonstrate tunable coupling strengths and mixing ratios among oscillators by exciting discrete and tunable GP modes in a graphene-integrated deep silver (Ag) grating. The mechanisms underlying the dynamic tunability of vibrational-polaritonic states are investigated through electromagnetic simulations and analytical methods, including a coupled-harmonic oscillator model and Hopfield coefficient analysis. This work addresses a critical gap in VSC research by enabling spectral access to and control over polaritonic states, paving the way for optically tunable molecular devices based on VSC.

## 2 Results and discussion

### 2.1 A graphene-integrated deep Ag grating for VSC

**Figure 1:**
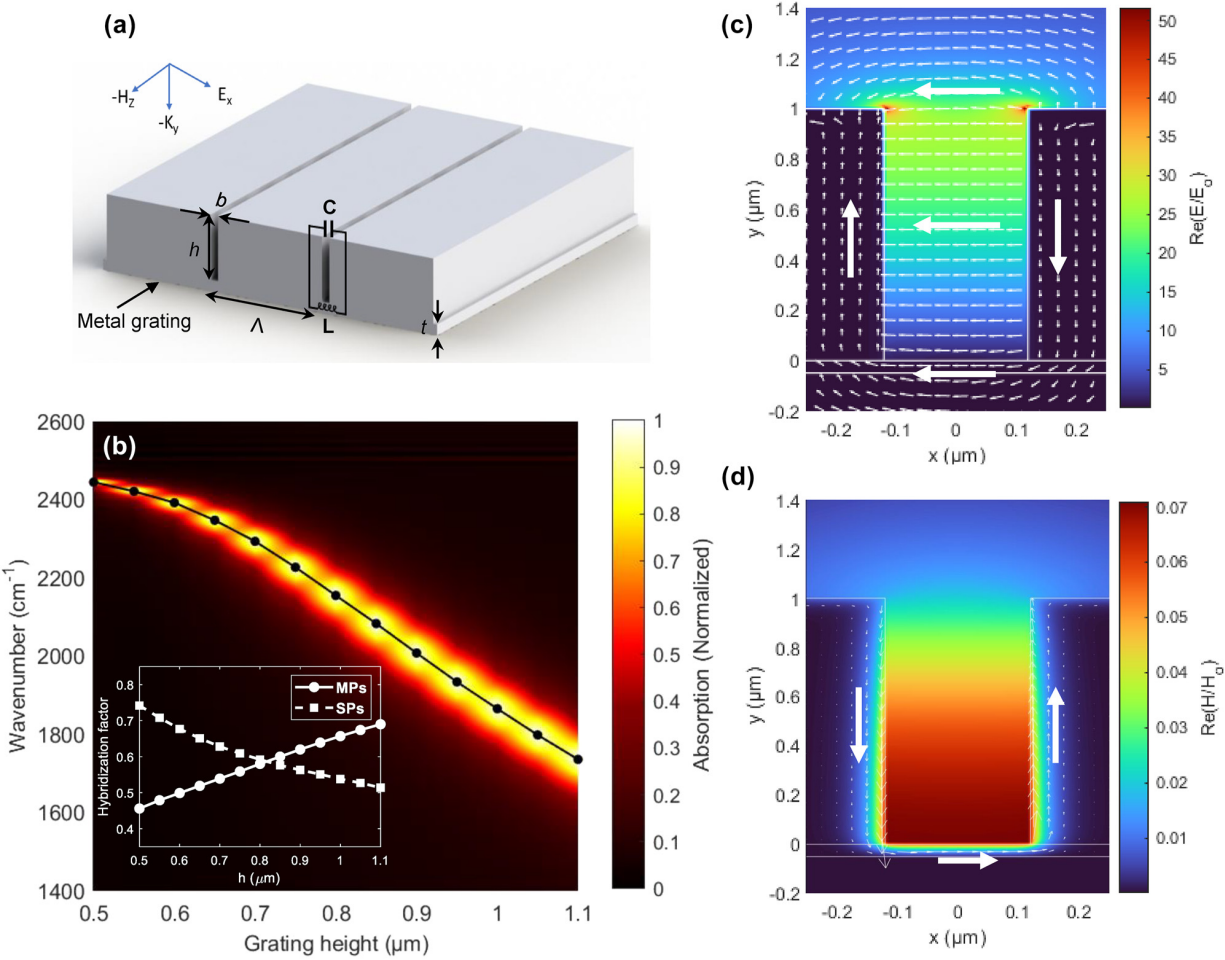
Deep Ag grating as an infrared resonator for VSC. (a) Schematic diagram of a deep Ag grating showing the structural parameters: period (Λ), trench gap (*b*), grating height (*h*), and thickness of the bottom metal film (*t*). Also shown is the equivalent LC circuit model, which comprises the metal inductance (L) and the capacitance of the air inside the trench (C). (b) Absorption intensity map of the deep Ag grating with Λ = 4 μm, *b* = 240 nm, *t* = 50 nm, and *h* varied from 0.5 to 1.1 μm. The solid black line with circular markers indicates resonance peaks calculated for mode hybridization between the MP and SP modes. The inset displays hybridization factors of the MP and SP modes as a function of *h*. (c) *E*- and (d) *H*-field intensity maps with vector field arrows for the deep Ag grating at *h* = 1.0 μm.

First, we optimized the structural parameters of a deep Ag grating to spectrally match its resonance frequency with the C=O stretching mode of PMMA at 1,730 cm^−1^ (Figure 1). The key structural parameters of the deep Ag grating include the period (Λ), trench gap (*b*), grating height (*h*), and the thickness of the bottom metal film (*t*) (Figure 1a). Within the spectral range of 1,400–2,600 cm^−1^, a single absorption peak was observed from the deep Ag grating upon excitation with transverse magnetic (TM) waves at normal incidence (Figure 1b and Figure S1a; for the grating with Λ = 4 μm, *b* = 240 nm, *t* = 50 nm, and *h* varied from 0.5 to 1.1 μm). As *h* increased from 0.5 to 1.1 μm, the absorption peak frequency shifted from 2,426 to 1,736 cm^−1^, while the full width at half maximum (FWHM; Γ_g_) broadened from 18 to 145 cm^−1^. Although spectral tuning could be achieved by varying Λ, *b*, and *t*, the most significant spectral tuning was achieved by adjusting *h*. Silicon (Si) was considered as a substrate. However, simulation results indicate that the substrate has a minor effect on modulating the absorption spectra of the deep Ag grating (see Supplementary Material). This is because MP modes create strong electric field confinement and circulation through an oscillating current around the trench gap, rather than through interaction with the substrate (further discussion is provided below).

To corroborate the origin of the strong infrared light absorption, the electric (*E*)- and magnetic (*H*)-field maps were generated for the deep Ag grating with *h* of 1.0 μm. At the resonance frequency of 1,865 cm^−1^, the *E*-field intensity was concentrated near the opening of the Ag grating, with *E*-field hot spots located at the edges of the trench opening (Figure 1c). The *E*-field vector arrows revealed a circulating pattern inside the trench gap, forming a loop around the grating opening. In contrast, the enhanced *H*-field was confined at the bottom of the Ag grating, exhibiting a single antinode within the trench gap (Figure 1d), indicative of the formation of the first-order MP mode [45]. This strong *H*-field confinement inside the trench gap induced antiparallel currents along the trench walls (arrows in Figure 1d), demonstrating the typical diamagnetic characteristic of the MP mode [46]. The enhanced electromagnetic fields in the deep Ag grating give rise to distinct absorption peaks in the mid-infrared spectral range, enabling effective coupling with molecular vibrations.

However, comprehensive electromagnetic simulations have revealed that the deep Ag grating supports not only MP modes but also surface plasmon (SP) modes. The *E*-field intensity map, simulated for the real part of the *y*-component, Re(*E*_*y*_), showed *E*-field distributions of the SP mode at the edges of the trench opening, indicated by + and − signs (Figure S1b). These distributions resemble those observed in metal groove structures supporting channel plasmon polaritons propagating along the tapered groove axis [47]. Furthermore, the deep Ag grating exhibited angle-dependent absorption spectra (Figure S1c), confirming the formation of the SP mode. The SP mode typically shows strong angle dependence, as observed in similar metal grating structures supporting surface plasmon polaritons [48]. In contrast, the MP mode is relatively angle independent due to its localized resonance nature [40].

Therefore, we calculated hybridization factors to analyze the hybridization between the MP and SP modes. The resonance wavelength of the MP mode was analytically derived using an equivalent LC circuit model – comprising the metal inductance (L) and the capacitance of the air inside the trench (C) (Figure 1a) [49]. Meanwhile, the maximum peak wavelength of the SP mode was determined from the SP dispersion relation expressed in terms of Λ [50]. The solid black line with circular markers in Figure 1b represents the hybridized MP and SP modes calculated for the deep Ag grating with varying *h*, showing excellent agreement with the simulated results (background intensity plot). The inset in Figure 1b plots the hybridization factors as a function of *h*, indicating that the MP dominates in gratings with larger *h*, while the SP mode is dominant in gratings with smaller *h*. Hereinafter, the hybridized MP and SP mode will be referred to as simply the *grating mode*. Details about the mode hybridization are provided in the Supplementary Material.

**Figure 2:**
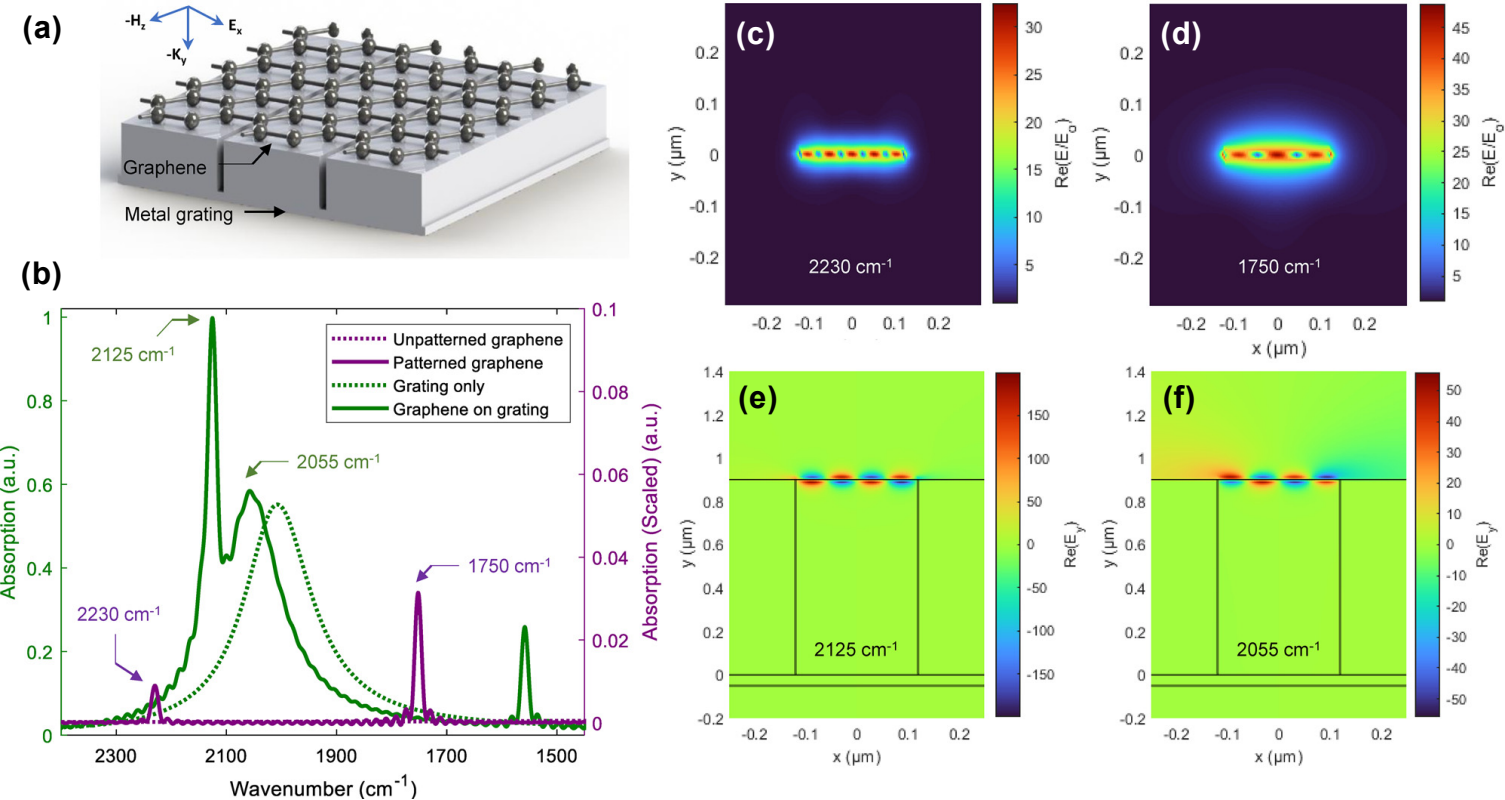
Graphene-integrated deep Ag grating. (a) Schematic diagram. (b) Absorption spectra of unpatterned graphene (dotted purple), patterned graphene ribbon with a width of 240 nm (solid purple), graphene-free deep Ag grating (dotted green), and graphene-integrated deep Ag grating (solid green). A chemical potential (*μ*) of 0.5 eV was applied to graphene in all cases. (c, d) *E*-field intensity maps of the patterned graphene ribbon at 2,230 and 1,750 cm^−1^, respectively. (e, f) Real part of the *y*-component *E*-field intensity maps of the graphene-integrated deep Ag grating at 2,125 and 2,055 cm^−1^, respectively, showing strong GP modes with opposite intensity nodes and antinodes.

Next, we investigated the interaction between the deep Ag grating and single-layer graphene in the graphene-integrated deep Ag grating (Figure 2). Generally, GP modes cannot be directly excited by unpatterned graphene due to the momentum mismatch between the incident light and the GP modes [51], as is shown by the dotted purple line in Figure 2b. However, when graphene was patterned into a graphene ribbon matching the grating parameters, *i.e.*, specifically, with the ribbon width equal to the width of the grating gap (*b* = 240 nm), distinct GP absorption peaks appeared at 2,230 and 1,750 cm^−1^ (solid purple). The excitation of GP modes led to an increased *E*-field intensity at the graphene ribbon surface, which was evident from the frequency-dependent *E*-field intensity nodes and antinodes [52] shown in Figure 2c and d.

What is notable, however, is that the absorption of light was further increased when *unpatterned* single-layer graphene was placed directly on top of the deep Ag grating. In this case, the sharp GP resonance mode induced an asymmetric Fano resonance line shape (solid green) superimposed on the otherwise broad grating mode (dotted green). This sharp GP resonance played a crucial role in enabling access to polaritonic states, as discussed in detail below. The enhanced *E*-field intensity near the opening of the trench gap in the deep Ag grating (shown in Figure 1c) efficiently coupled to the GP modes in the graphene-integrated deep Ag grating, resulting in a strongly enhanced *E*-field tightly confined to the graphene surface (Figure 2e and f). It is reported that GPs are confined to volumes on the order of ∼10^6^ times smaller than the free-space wavelength [53], leading to estimated *V*_*m*_ in the range of ∼0.1 μm^3^ or less for graphene ribbons in the mid-IR [42], [54]. We expect that the *V*_*m*_ of our graphene-integrated deep Ag grating is comparable to this value, since unpatterned single-layer graphene supports highly confined GP modes on top of the deep Ag grating similar to those of the patterned graphene ribbons. This volume is significantly smaller than that of a polymer-filled Fabry–Pérot cavity, which typically ranges from 70 to 300 μm^3^ in the mid-IR [12], [55]. Since the coupling strength of VSC (*g*) is inversely proportional to $\sqrt{V_{m}}$ [56], this substantially reduced *V*_*m*_ enhances the interaction between light and matter – in this case, between the graphene-integrated deep Ag grating and PMMA.

### 2.2 Strong coupling between grating modes and molecular vibrations controlled by graphene plasmons

**Figure 3:**
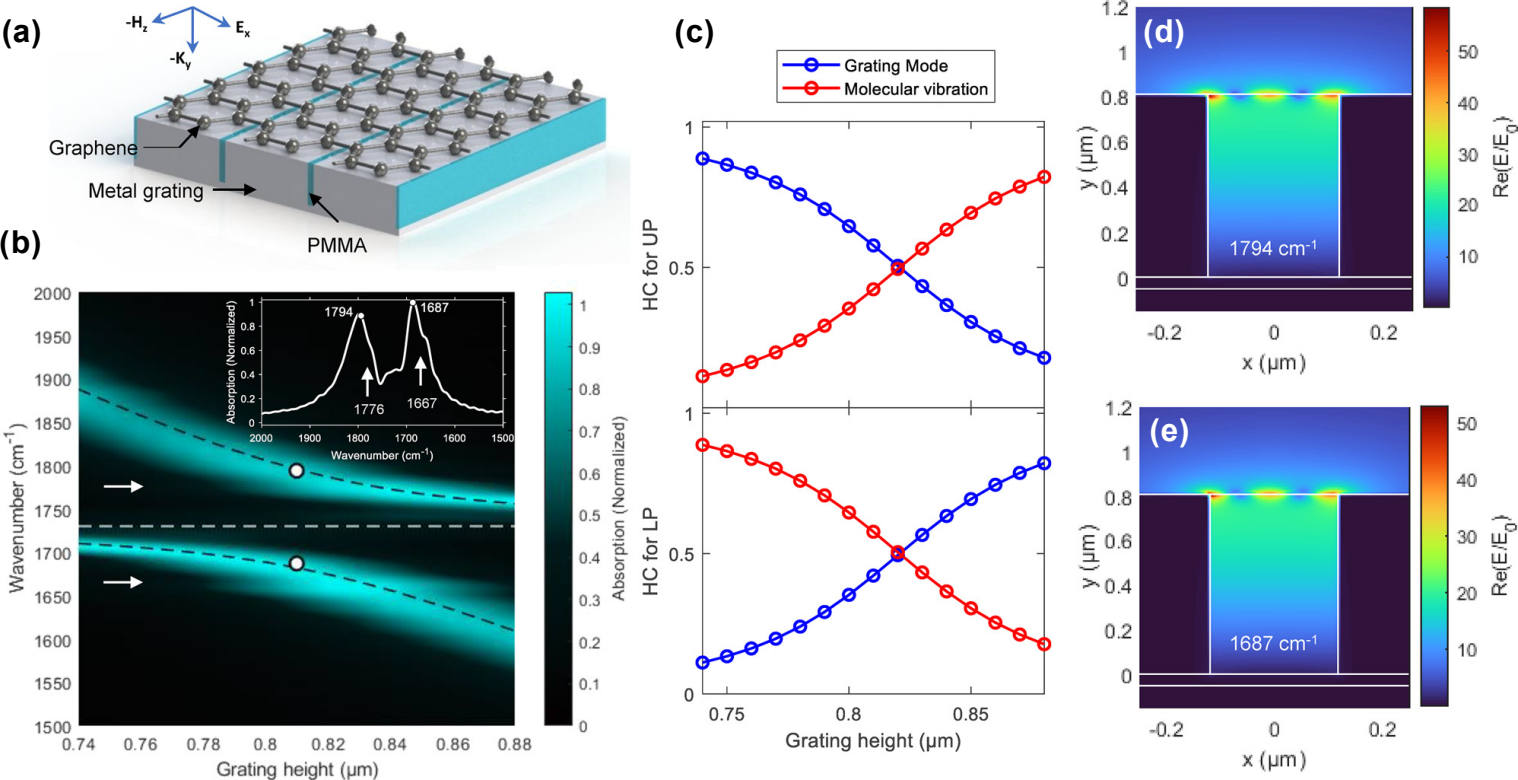
VSC between grating modes and molecular vibrations. (a) Schematic diagram of the graphene-integrated deep Ag grating with PMMA-filled trench gaps. (b) Absorption intensity map for grating parameters Λ = 4 μm, *b* = 240 nm, *t* = 50 nm, and varying *h* from 0.74 to 0.88 μm at *μ* = 0.5 eV. The CHO model fit (black dashed lines) suggests ℏΩ_R_ of 113 cm^−1^. A white dashed horizontal line indicates the frequency of the C=O stretching mode of PMMA at 1,730 cm^−1^, while white arrows mark the frequencies of the GP modes at 1,776 and 1,667 cm^−1^. The inset shows an absorption spectrum of the graphene-integrated deep Ag grating with *h* = 0.81 μm. Arrows indicate GP modes at 1,776 and 1,667 cm^−1^. (c) Hopfield coefficients (HC) for the upper (UP, top) and lower polaritonic modes (LP, bottom), representing contributions from the grating mode (blue) and molecular vibration (red), respectively. (d, e) *E*-field intensity maps of the grating at peak absorption frequencies of the UP (1,794 cm^−1^) and LP mode (1,687 cm^−1^).

Then, we investigated the role of discrete GP resonances in the spectral modulation of polaritonic states. First, we examined a graphene-integrated deep Ag grating with the trench gaps filled with PMMA (Figure 3). By varying *h*, the grating mode was tuned to sweep through the C=O stretching vibration of PMMA at 1,730 cm^−1^, resulting in clear peak splitting in the absorption spectra (Figure S2a). This splitting resulted in the formation of UP and LP states relative to the C=O stretching vibration indicated by the horizontal dashed line in Figure 3b. The anticrossing behavior observed in the *h*-dependent absorption spectra was quantitatively analyzed using a coupled-harmonic oscillator (CHO) model represented by a 2 × 2 matrix Hamiltonian (see Supplementary Material for details). The model fit (black dashed lines) closely matched the simulated data (background intensity plot) in Figure 3b, yielding a Rabi splitting energy (ℏΩ_R_) of 113 cm^−1^. Importantly, the obtained ℏΩ_R_ exceeds the average FWHM of both the uncoupled grating mode (Γ_g_ = 134 cm^−1^; Figure S2b) and the PMMA molecular vibration (Γ_PMMA_ = 25 cm^−1^; Figure S2c), satisfying the criterion for strong coupling [57].

The photonic (*i.e.*, grating mode) and material (*i.e.*, PMMA molecular vibration) contributions to the UP and LP modes were further characterized by calculating the Hopfield coefficients (Figure 3c). The UP mode exhibited a more material-like character at larger values of *h* and a more photon-like character at smaller *h*, with the opposite trend observed for the LP mode. At *h* = 0.82 μm, both UP and LP modes showed equal mode mixing, indicating strong hybridization between the grating modes and molecular vibrations. Contributions from the GP mode were negligible and thus excluded from both the CHO model fit and the Hopfield coefficient analysis.

Figure 3d and e shows the *E*-field intensity maps of the PMMA-filled, graphene-integrated deep Ag grating (*h* = 0.81 μm) at the frequencies corresponding to the UP (1,794 cm^−1^; Figure 3d) and LP mode (1,687 cm^−1^; Figure 3e). Additional *E*-field intensity maps at other characteristic frequencies are provided in Figure S3. Compared to the PMMA-filled control grating without graphene (Figure S4), the *E*-field intensity was enhanced due to the excitation of GP modes in Figure 3d and e. The *E*-field intensity was tightly confined to the graphene surface, enabling VSC with molecules located in regions of increased *E*-field intensity. However, when the trench gap of the deep Ag grating was filled with PMMA, the imaginary part of the GP dispersion increased due to the high dielectric constant of PMMA. This led to a decrease in the propagation length of the GP mode by approximately 1.5-fold compared to the air-filled deep Ag grating (see Supplementary Material for details). Consequently, the GP mode was suppressed, appearing only as faint streaks in the UP and LP branches (indicated by white arrows in Figure 3b) and as subtle kinks in the absorption spectrum at 1,776 and 1,667 cm^−1^ (inset of Figure 3b). After all, the overall results were very similar to those of the PMMA-filled control grating without graphene, which exhibited a similar ℏΩ_R_ value of 111 cm^−1^ (Figure S4b). The GP mode showed a negligible effect on modulating polaritonic states in the PMMA-filled deep Ag grating.

**Figure 4:**
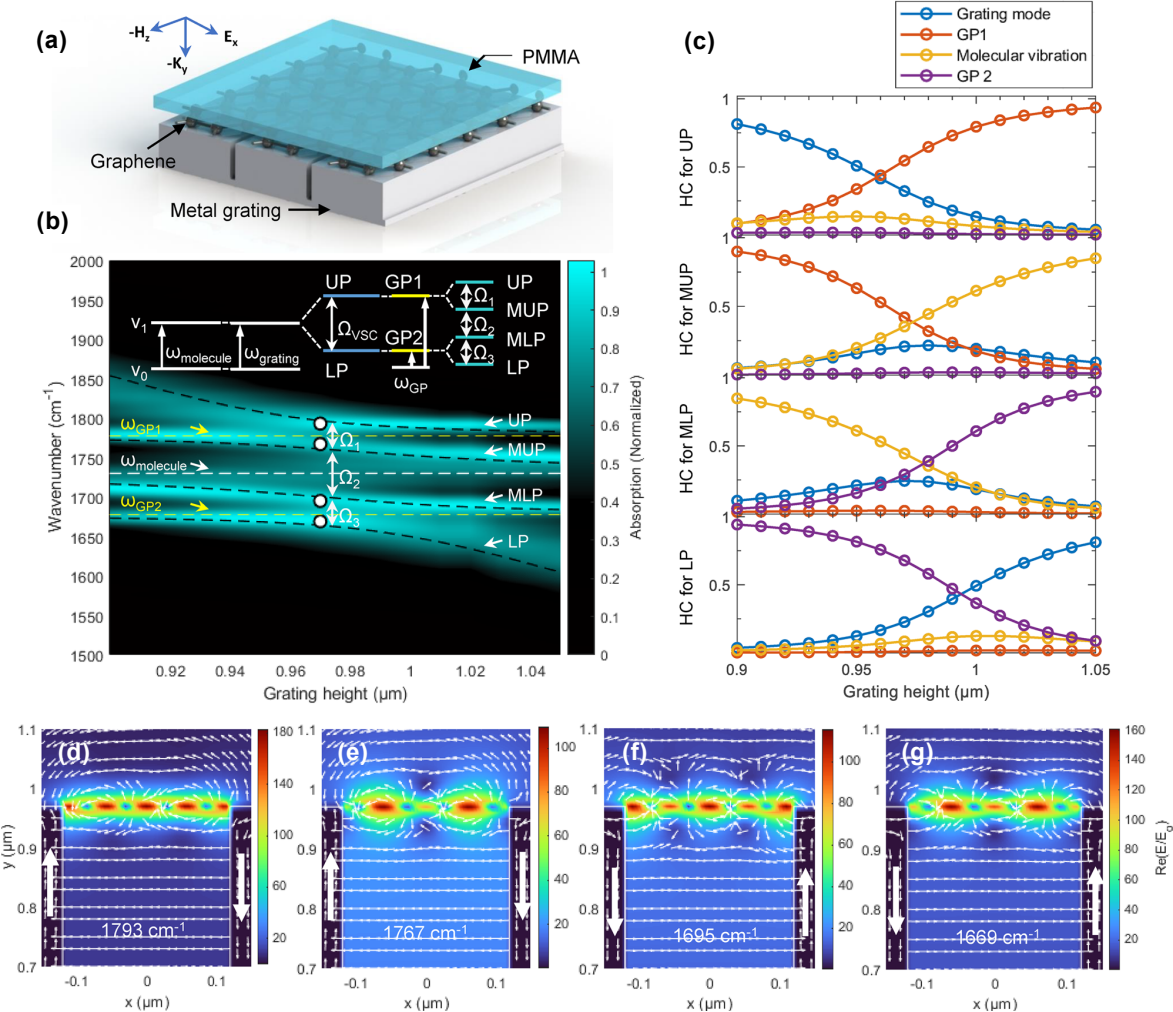
VSC controlled by graphene. (a) Schematic diagram of the graphene-integrated Ag grating with a thin PMMA slab placed on top of the graphene layer. (b) Absorption intensity map for grating parameters Λ = 4 μm, *b* = 240 nm, *t* = 50 nm, and varying *h* from 0.90 to 1.05 μm at *μ* = 0.5 eV. The CHO model fit (black dashed lines) suggests *g*_1_ = 0.003 eV between UP and MUP, *g*_2_ = 0.005 eV between MUP and MLP, and *g*_3_ = 0.003 eV between MLP and LP. A white dashed horizontal line indicates the C=O stretching frequency of PMMA at 1,730 cm^−1^, while yellow dashed lines mark the GP mode frequencies at 1,778 (GP1) and 1,678 cm^−1^ (GP2). Inset: Energy diagram illustrating the vibrational transition (*ν*_0_–*ν*_1_) of a molecule (*ω*_molecule_) resonant with a cavity mode (*ω*_grating_), forming hybrid polaritonic states UP and LP separated by Ω_VSC_. Two discrete GP modes (GP1 and GP2) further split each UP and LP mode into UP, MUP, MLP, and LP, resulting in three Rabi splitting values of Ω_1_, Ω_2_, and Ω_3_. (c) Hopfield coefficients for UP, MUP, MLP, and LP modes, showing mode mixing contributions from the grating mode (blue), GP1 (orange), molecular vibration (yellow), and GP2 (purple). (d–g) *E*-field intensity maps at the peak frequencies of UP (d, 1,793 cm^−1^), MUP (e, 1,767 cm^−1^), MLP (f, 1,695 cm^−1^), and LP (g, 1,669 cm^−1^) at *h* = 0.97 μm (indicated by white dots in Figure 4b).

Therefore, we investigated a graphene-integrated deep Ag grating with a 300 nm-thick PMMA slab placed on top of the graphene layer (Figure 4a). When the trench gaps of the grating were left filled with air, the GP mode became more tightly confined to the graphene surface near the grating openings, precisely where VSC occurs with PMMA. These strong GP modes enabled effective modulation of the vibrational-polaritonic states, as reflected by the varied ℏΩ_R_ values and mixing ratios between the individual oscillators. First, similar to the PMMA-filled grating, the absorption intensity plot in Figure 4b demonstrates strong coupling between the grating modes and molecular vibrations, resulting in major spectral splitting around the C=O stretching mode frequency of PMMA at 1,730 cm^−1^ (indicated by the white dashed line). These inherent upper and lower polaritonic states are broad resonances, with their spectral positions determined mainly by fixed system parameters such as *h*, *b*, Λ, and PMMA thickness. As such, there is only limited spectral access to individual polaritonic modes.

However, when graphene is integrated into the grating in this configuration, each UP and LP mode further splits into two distinct branches due to sharp GP modes located at 1,778 and 1,678 cm^−1^ (each GP mode is designated as GP1 and GP2 modes, respectively; yellow dashed lines), giving rise to two middle polaritonic modes of middle-upper polariton (MUP) and middle-lower polariton (MLP) modes (see Figure S5 for the full *h*-dependent absorption spectra). The integration of graphene provides the deep Ag grating with spectral access to the upper and lower polaritonic states via excitation of discrete GP modes. Importantly, the frequencies of the GP modes can be tuned by adjusting the chemical potential of graphene or by varying the number of graphene layers as will be discussed in the following. Therefore, graphene plasmons enable dynamic modulation of otherwise static polaritonic states thanks to their sharp spectral features, mode selectivity, and tunability.

Coupling to multiple discrete GP modes results in multiple ℏΩ_R_ values as represented in the inset of Figure 4b. A CHO model using a 4 × 4 matrix Hamiltonian (black dashed lines in Figure 4b) yielded coupling strengths of *g*_1_ = 0.003 eV between UP and MUP, *g*_2_ = 0.005 eV between MUP and MLP, and *g*_3_ = 0.003 eV between MLP and LP. The model fit showed excellent agreement with the simulated data in Figure 4b. Through a closer inspection of Figure 4b, one may observe that while Ω_1_ and Ω_3_ exhibit the minimum energy separation between adjacent branches, Ω_2_ remains almost constant across *h*. Notably, the evolution of Ω_2_ more closely resembles the spectral shape of the UP and LP branches shown in Figure 3b, suggesting that different mechanisms are at play in the multimode coupling. We also note that although the minimum spectral splittings are obtained at different values of *h* (*i.e.*, Ω_1_ at *h* = 0.97 μm and Ω_3_ at *h* = 0.98 μm), the Ω values at a constant *h* of 0.97 μm are indicated in Figure 4b. This is because, in practice, a single deep Ag grating with a given *h* would be used to determine the Ω values between branches.

To better understand the multimode coupling mechanisms in our system, we evaluated whether the strong coupling condition is fulfilled (see Supplementary Material). While various strong coupling criteria exist [58], [59], we adopted the criterion that defines strong coupling as Ω being greater than the average linewidth of the oscillators involved, which is most universal. This criterion is clearly fulfilled for the coupling between the MUP and MLP branches, indicating that strong coupling is achieved between the grating mode and molecular vibrations. However, it is not reached for the UP-MUP and MLP-LP couplings. The two discrete GP modes, therefore, exhibit only weak coupling, resulting in the observed spectral splits within the UP-MUP and MLP-LP regions. We emphasize that the opportunity presented by graphene-integrated deep Ag grating structures lies in their spectral accessibility to polaritonic states via discrete GP modes – even when the strong coupling criterion is not fully satisfied. Nevertheless, with realistic design adjustments, particularly by tuning the FWHM of the grating mode, our system could approach the strong coupling regime for all oscillators.

The Hopfield coefficients were calculated to quantify the contributions of each mode in the coupled system, resulting in the mixing ratios among the grating mode, GP1, molecular vibrations, and GP2 (Figure 4c). For the UP mode at *h* = 0.96 μm, the mixing ratio was 0.41:0.44:0.13:0.02, indicating dominant contributions from the grating mode and GP1, with minor mixing from molecular vibrations and GP2. The MUP mode at *h* = 0.97 μm exhibited significant mixing among GP1, molecular vibrations, and the grating mode, with a ratio of 0.21:0.42:0.35:0.02. At smaller *h* values, MUP was primarily dominated by GP1, but as the grating mode approached the molecular vibration frequency with increasing *h*, the contribution from the molecular vibration grew. The contribution from GP2 was negligible due to its spectral distance from MUP. Similarly, the MLP mode at *h* = 0.98 μm showed substantial mixing between GP2, molecular vibrations, and the grating mode, with a ratio of 0.24:0.02:0.35:0.39. The GP1 mode had little influence on MLP because of spectral distance. These Hopfield coefficients for MUP and MLP confirm strong coupling among the three oscillators and highlight the crucial role of the GP modes in controlling mode mixing and polaritonic states. Finally, the LP mode at *h* = 0.99 μm exhibited mode mixing primarily between GP2 and the grating mode, with minor contributions from molecular vibrations and GP1. The mixing ratio for LP was 0.40:0.02:0.11:0.47. Notably, effective mode mixing – especially at the crossing points – occurs at different *h* values for each of the UP, MUP, MLP, and LP modes, indicating complex mode interactions. A detailed description of the 4 × 4 CHO model fit and the complete set of mixing coefficients are provided in the Supplementary Material.

The *E*-field intensity distribution maps in Figure 4d–g provide insights into the strong coupling between grating modes and molecular vibrations, as well as the polaritonic states controlled by the GP modes. At *h* = 0.97 μm (indicated by white dots in Figure 4b), the *E*-field intensity was concentrated along the graphene layer suspended within the air gap of the grating across all frequencies corresponding to (d) the UP, (e) MUP, (f) MLP, and (g) LP modes. Strong GP modes manifested as distinct *E*-field nodes and antinodes, causing the electric vector fields to circulate at the interface between PMMA, graphene, and the air gap of the deep Ag grating. These strong GP modes enabled the *E*-field intensity to extend into the PMMA layer, thereby enhancing the coupling between the grating modes and molecular vibrations. Interestingly, the *E*-field vector arrows exhibited opposite circulation directions between the UP (clockwise) and LP mode (counter-clockwise) along the grating walls. In contrast, at the frequency of the PMMA C=O stretching mode (1,730 cm^−1^, Figure S6e), the GP mode vanished, leaving *E*-field intensity hotspots localized at the edges of the trench opening, similar to the pattern observed in the bare grating structure (Figure 1c).

These results demonstrate that strong, discrete GP modes enable spectral access to vibrational-polaritonic states, effectively tuning the ℏΩ_R_ values and mixing ratios between individual oscillators when a PMMA slab is placed on top of the graphene layer. Previous research in VSC has primarily focused on single-mode cavity systems to achieve hybridization between molecular vibrations and confined electromagnetic fields [12], [60], [61]. Meanwhile, graphene plasmonics has been extensively explored for its tunable mid-infrared resonances and strong field confinement [43], [53]. However, the integration of multiple discrete GP modes to simultaneously couple with molecular vibrations for multilevel polaritonic splitting remains largely unexplored. Our results bridge this gap by leveraging a graphene-integrated deep Ag grating that supports multiple discrete GP modes, enabling multimode VSC within a single device architecture. This is possible due to graphene plasmon modes, which provide spectral resolution, mode selectivity, and tunability. Beyond the mere formation of vibrational-polaritonic states – which has been shown to modify chemical reactivity [13], [14] – our findings further demonstrate that these states can be controlled by multiple discrete GP modes, potentially influencing bond-specific chemical reactions and VSC-modified molecular properties through tunable light–matter interactions.

### 2.3 Tunability of VSC with graphene

**Figure 5:**
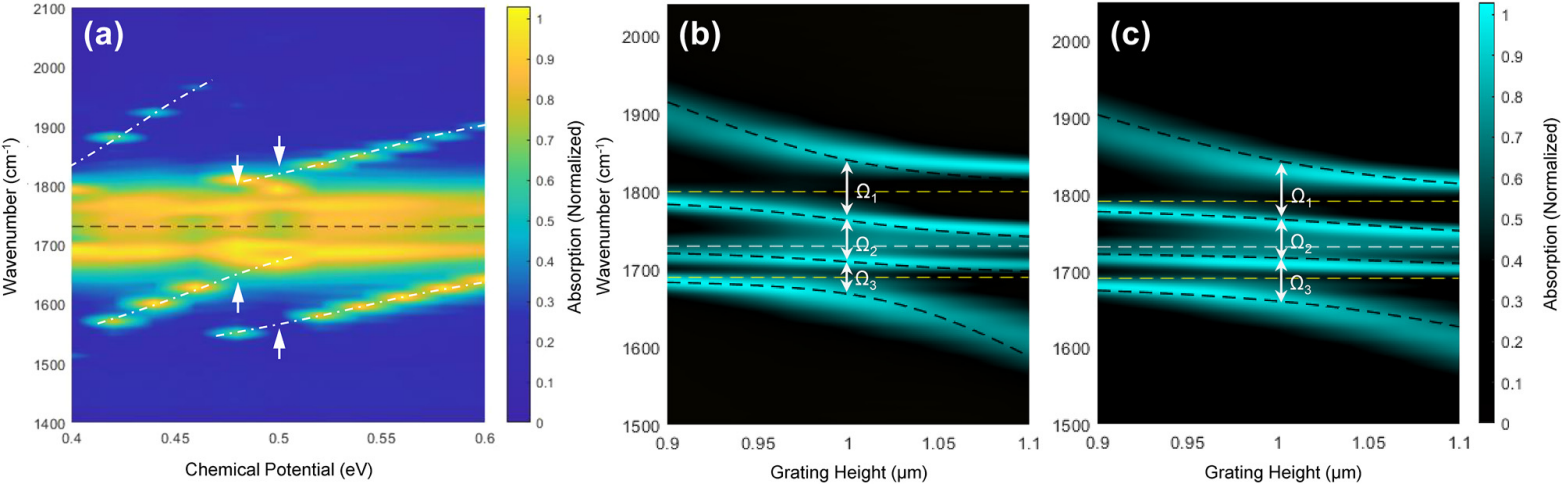
Tunability of VSC with graphene. (a) Absorption intensity map with *µ* varied from 0.4 to 0.6 eV in 0.02 eV increments. Dashed dot lines indicate the GP modes shifting linearly when off-resonant with the polaritonic states (0.40–0.46 eV and 0.52–0.60 eV). White arrows highlight the GP modes shifting abruptly when resonant with the polaritonic states at 0.48 and 0.50 eV. A black dashed horizontal line indicates the C=O stretching frequency of PMMA at 1,730 cm^−1^. Absorption intensity maps of the deep Ag grating with (b) double-layer and (c) triple-layer graphene. The CHO model fit (black dashed lines) and ℏΩ_R_ values are indicated. A white dashed horizontal line marks the C=O stretching frequency of PMMA, and yellow dashed lines indicate the frequencies of the GP modes.

Building on the ability to modulate polaritonic states using graphene, we explored further tunability of VSC by varying (1) the chemical potential applied to graphene (*µ*) and (2) the number of graphene layers integrated into the grating. First, a *μ*-dependent absorption intensity map was obtained for the graphene-integrated deep Ag grating with a PMMA layer placed on top of the graphene layer (Figure 5a). As *µ* was varied from 0.4 to 0.6 eV in increments of 0.02 eV, the frequency of the GP mode shifted linearly when the GP modes were off-resonant from the polaritonic states, specifically within the ranges of 0.40–0.46 eV and 0.52–0.60 eV (dash-dot lines in Figures 5a and S7). These off-resonance results are consistent with the known linear relationship between the plasmon resonance frequency and the Fermi energy of graphene ribbons [62], [63].

However, when the GP modes approached resonance with the polaritonic states at 0.48 and 0.50 eV (indicated by arrows), their frequencies shifted abruptly, causing spectral dips in the grating mode. This phenomenon can be explained by “frequency pushing,” which is the frequency shift of a cavity mode away from its original position as it approaches a material resonance [64]. Due to the dispersive interaction with the material resonance, the frequency shift grows larger, reaching a maximum near the so-called exceptional point, after which the single peak splits into two, corresponding to the formation of polaritonic states. In our system, the observed frequency shifts as the GP mode approached the polaritonic states by tuning *µ*, indicating that the hybrid light–matter polaritonic states inherit the dispersive properties of the material resonance. These results demonstrate that not only can the GP mode frequency be dynamically tuned by varying *µ* but also that the GP mode actively couples to the vibrational-polaritonic states, thereby modulating what would otherwise be static polaritonic modes.

In addition, we investigated the modulation of polaritonic states by using multilayer graphene integrated with the deep Ag grating (Figures 5b, c and S8). For double-layer graphene at *µ* = 0.5 eV, the GP modes shifted to higher wavenumbers compared to single-layer graphene, with GP1 moving from 1,778 to 1,800 cm^−1^ and GP2 from 1,678 to 1,690 cm^−1^. This shift resulted in strong coupling occurring at a higher *h* of 1.0 μm. With triple-layer graphene, strong coupling was achieved at a lower *μ* of 0.35 eV, where GP1 and GP2 appeared at 1,790 and 1,690 cm^−1^, respectively. By adjusting the values of *h* and *μ* to tune the GP modes, multilevel polaritonic splitting was consistently observed in the deep Ag grating integrated with either double- or triple-layer graphene.

The CHO model fits showed excellent agreement with the simulation results in Figure 5b and c (black dashed lines). For the grating with double-layer graphene, the coupling strength was 0.006 eV between the UP and MUP, 0.005 eV between MUP and MLP, and 0.004 eV between MLP and the LP. Compared to the single-layer graphene, the coupling strengths increased notably, especially between UP and MUP as well as between MLP and LP. For the grating with triple-layer graphene, coupling strengths were further increased and were more evenly distributed, resulting in 0.006 eV (UP-MUP), 0.005 eV (MUP-MLP), and 0.006 eV (MLP-LP). The increased coupling strength is a direct consequence of the enhanced absorption associated with a greater number of graphene layers, as demonstrated for stacked graphene layers in previous studies [43]. Additionally, as the number of graphene layers increases, the resonance frequency of the GP mode shifts, which in turn modifies the coupling strengths observed in each ℏΩ_R_ value. The mixing coefficients indicated significant mode mixing among the grating mode, molecular vibrations, and the two GP modes for both double- and triple-layer graphene cases (Figure S8c and d). These results highlight the unique capability of the graphene-integrated deep Ag grating to control the coupling strength and mode mixing between different oscillators, offering a robust platform for studying hybrid half-light, half-matter polaritonic states.

## 3 Conclusions

We have demonstrated that the graphene-integrated deep Ag grating serves as a promising infrared resonator to effectively induce and modulate vibrational-polaritonic states. The vibrational-polaritonic states arise from the strong coupling between the grating modes of the deep Ag grating and the molecular vibrations of PMMA. The deep Ag grating structure localizes the *E*-field intensity at the trench openings, facilitating the excitation of strong and discrete GP modes that directly influence the coupling strength and mode mixing among the coupled oscillators. Unlike previous strong coupling studies that primarily focus on maximizing Rabi splitting through equal mode contributions, the graphene-integrated deep Ag grating enables exploration of polariton-modulated material properties with tunable light–matter interactions. In addition, the graphene-integrated deep Ag grating allows positioning of molecular absorbers at the maximum optical field and enables probing of strong coupling with molecules placed outside the grating in an open-cavity design, [65] which further allows access to external chemistry and measurements using near-field probes. Future research directions include dynamic VSC studies to monitor molecular property changes via electrical biasing of graphene, as well as integrating other types of two-dimensional materials – such as transition metal dichalcogenides or topological insulators – into the deep Ag grating structure.

## 4 Methods

We used the finite-difference time-domain (FDTD) method to simulate the deep Ag grating structure integrated with molecular layers and graphene, employing Ansys Lumerical v2019. The model was constructed in a 2D environment along the *x*–*y* plane, assuming infinite uniformity in the *z*-direction with respect to material properties. In the *x*-direction, periodic boundary conditions (BC) were applied for normal incidence, while Bloch BCs were used for oblique incidence angles. For the *y*-direction, perfectly matched layer (PML) BCs were implemented to absorb outgoing waves and prevent reflections. An auto nonuniform mesh with a mesh accuracy level of 4 was used throughout all simulations. Additionally, a uniform mesh refinement of 5 nm in the *x*-direction and 10 nm in the *y*-direction was applied across the entire structure geometry to ensure numerical precision. A plane wave source with a polarization angle of 0° was used to excite transverse magnetic (TM) polarized light. Two linear monitors oriented along the *x*-axis captured reflected and transmitted light, positioned above the top surface and beneath the grating, respectively. To analyze the *E*- and *H*-field intensities, a frequency-domain field profile monitor was used. Optical constants for Ag [66] and PMMA [67] were obtained from the literature.

A 2D graphene sheet was modeled with a scattering rate of Γ = 7.81 × 10^11^ s^−1^ and corresponding energy, *E* = *ℏ*Γ = 5.14 × 10^−4^ eV at a temperature of *T* = 300 K. The graphene sheet was oriented normal to the *Y*-axis in the simulation. The optical conductivity (*σ*) of graphene was calculated using the Kubo formula as a function of angular frequency (*ω*), chemical potential (*μ*), Γ, and *T*. The total optical conductivity $\sigma \left(\omega ,\mu ,\Gamma ,T\right)$ consists of contributions from both intraband and interband electronic transitions [68], expressed as: $\sigma \left(\omega ,\mu ,\Gamma ,T\right)=\sigma_{\text{intra}}\left(\omega ,\mu ,\Gamma ,T\right)+\sigma_{\text{inter}}\left(\omega ,\mu ,\Gamma ,T\right)$ .

## Supplementary Material

Supplementary Material Details
