# Supplementary material for: Vibrational strong coupling modulated by graphene plasmons in deep metal grating structures
Source: Nanophotonics. 2025 Sep 15;14(19):3169–80. doi: 10.1515/nanoph-2025-0275 (PMC12455289; doi:10.1515/nanoph-2025-0275)
Supplement: Supplementary file 1 — Supplementary Material Details [file j_nanoph-2025-0275_suppl_001.docx]

**Vibrational Strong Coupling Modulated by Graphene Plasmons in Deep Metal Grating Structures**

Md Faysal Hossain and Wonmi Ahn*

UNAM – National Nanotechnology Research Center and Institute of Materials Science and Nanotechnology, Bilkent University, Ankara, Türkiye

*E-mail: wonmi.ahn@unam.bilkent.edu.tr

**Supplementary material**

**Contents**

- Effects of the substrate on the deep Ag grating structure.
- Hybridization between magnetic polariton and surface plasmon modes in a deep Ag grating structure.
- Coupled harmonic oscillator model.
- Dispersion relation and propagation length of graphene plasmons.
- Supplementary Figures.

Figure S1. A bare deep Ag grating structure.

Figure S2. Absorption spectra of a graphene-integrated deep Ag grating with PMMA filled in the trench gap.

Figure S3. *E*-field intensity maps of a graphene-integrated deep Ag grating with PMMA filled in the trench gap.

Figure S4. Graphene-free deep Ag grating with PMMA filled in the grating gap.

Figure S5. Graphene-integrated deep Ag grating with a PMMA layer on top of graphene.

Figure S6. *E*-field intensity maps of the graphene-integrated Ag grating with a PMMA layer on top of graphene.

Figure S7. Tunable VSC by varying the chemical potential applied to graphene.

Figure S8. Tunable VSC by varying the number of graphene layers.

**Effects of the substrate on the deep Ag grating structure.**

Silicon (Si) was chosen as a substrate due to its infrared transparency, especially in its undoped form. However, the influence of the substrate was found to be negligible because light is primarily absorbed within the deep Ag grating rather than transmitted through the Si substrate. The absorption and transmission spectra are shown below for the graphene-integrated deep Ag grating with a PMMA slab placed on top of the graphene layer. The absorption spectra remained identical irrespective of the presence of the Si substrate, while the transmission spectrum showed that the deep Ag grating structure transmits almost no light even without the Si substrate. This behavior is attributed to the unique properties of magnetic polaritons, which generate strong electric field confinement and circulation through an oscillating current around the trench gap, rather than relying on interaction with the substrate.

Absorption and transmission spectra of the graphene-integrated deep Ag grating with a PMMA slab placed on top of the graphene layer (Λ = 4 μm, *b* = 240 nm, *t* = 50 nm, and *h* = 0.97 μm at *μ* = 0.5 eV). The blue and red lines represent the absorption spectra without and with a Si substrate, respectively, while the yellow line shows the transmission spectrum without a Si substrate.

**Hybridization between magnetic polariton and surface plasmon modes in a deep Ag grating structure.**

The hybridization between the magnetic polariton (MP) and surface plasmon (SP) modes was analyzed by calculating hybridization factors for each mode. First, we referred to Zhang’s study [^[[1]](#endnote-1)^] to calculate the resonance wavelength of the MP mode ($\lambda_{MP}$). An equivalent LC circuit model was applied to the deep Ag grating, treating the air surrounding the grating as a capacitor and the metal as a conductor. The total impedance, $Z(\omega)$, is expressed:

$Z\left( \omega\right)=i\omega\left( L_{k}+L_{m}-\frac{1}{\omega^{2}C} \right)$ (S1)

where $L_{k}$ and $L_{m}$ are the kinetic and mutual inductance, respectively, and $C$ is the capacitance of the air inside the trench. Here, $L_{k}$ and $L_{m}$ represent the frequency-dependent complex impedance of the metal (Equation S2) and the impedance caused by closely placed grating walls (Equation S3), respectively.

$L_{k}=-\frac{2h + b}{\varepsilon_{0} \omega^{2} l \delta}\frac{\varepsilon'}{\left( \varepsilon'^{2} + \varepsilon''^{2} \right)}$ (S2)

$L_{m}=\mu_{0}\frac{\mathrm{hb}}{l}$ (S3)

where $h$ is the grating height, $b$ is the trench width, and $l$ is the length of the grating in *z*-direction. $\varepsilon^{'}$ and $\varepsilon^{''}$ are the real and imaginary parts of the metal’s dielectric function, respectively. $\varepsilon_{0}$ and $\mu_{0}$ are the electric permittivity and permeability of vacuum, respectively. The parameter $\delta$ is the penetration depth of the electric field, defined as $\delta=\lambda/2\pi\kappa$, where $\kappa$ is the extinction coefficient of the metal. Following Zhang’s approach, an effective numerical factor $k$ = 0.5 was used to account for the charge density distribution along the trench gap, which increases from zero at the bottom to a maximum near the trench opening. This assumption is consistent with our simulation results (**Figure 1c**). The capacitance $C$ was then calculated as:

$C=k\varepsilon_{0}hl/b$ (S4)

The MP resonance condition is obtained when $Z\left( \omega\right)=0$ in Equation S1. Therefore, the resonance wavelength of the MP mode ($\lambda_{MP}$) is given by:

$\lambda_{MP}=2\pi c\sqrt{\left( L_{k}+L_{m} \right)C}$ (S5)

where $c$ is the speed of light.

We also calculated the resonance wavelength of the SP modes ($\lambda_{SP}$) at normal incidence using the SP dispersion relation expressed in terms of the period of the grating ($\Lambda$) [^[[2]](#endnote-2)^].

$\lambda_{SP}=\Lambda\sqrt{\frac{\epsilon_{1}\epsilon_{2}}{\epsilon_{1}+\epsilon_{2}}}$ (S6)

where $\epsilon_{1}$ and $\epsilon_{2}$ are the dielectric constants of the interface medium and metal, respectively.

Finally, the hybridized resonance wavelength ($\lambda_{Hybrid}$) was obtained as a weighted sum of the SP and MP resonance wavelengths:

$\lambda_{Hybrid}=x\cdot\lambda_{SP}+y\cdot\lambda_{MP}$ (S7)

where $x$ and $y$ are the hybridization factors for the SP and MP modes, respectively. The values of $\lambda_{MP}$ and $\lambda_{SP}$ were calculated from Equations S5 and S6, respectively, which correspond to the cases when $x=0$ and $y=0$ in Equation S7. We then determined $x$ and $y$ by fitting to the simulated resonance wavelengths, revealing the hybridized MP and SP modes. The inset in **Figure 1b** shows the variation of $x$ and $y$ as a function of *h*.

**Coupled harmonic oscillator model.**

The upper and lower polaritonic modes observed in the *h*-dependent absorption spectra were fitted using the coupled-harmonic oscillator (CHO) model. For the PMMA-filled deep Ag grating, the effect of graphene plasmon (GP) modes on the coupling was negligible (**Figure 3**). Therefore, the coupling was described by a 2 x 2 matrix Hamiltonian representing the interaction between the grating mode and the carbonyl (C=O) stretching mode of poly(methyl methacrylate) (PMMA):

$\left( \begin{matrix} E_{GM}\left( h \right) & g \\ g & E_{m} \end{matrix} \right) \left( \begin{matrix} \begin{matrix} \alpha\\ \beta\end{matrix} \end{matrix} \right) = E_{UP, LP} \left( \begin{matrix} \begin{matrix} \alpha\\ \beta\end{matrix} \end{matrix} \right)$ (S8)

The eigenvalues of this matrix yield the upper and lower polaritonic energies of $E_{UP}$ and $E_{LP}$ with the coupling strength ($g$) [^[[3]](#endnote-3)^], [^[[4]](#endnote-4)^].

$E_{UP,LP}=\frac{E_{GM}+E_{m}}{2}\pm\frac{1}{2}\sqrt{\left( E_{GM}-E_{m} \right)^{2}+{4g}^{2}}$ (S9)

Here, $E_{GM}\left( h \right)$ and $E_{m}$ represent the resonance energies of the *h*-dependent grating mode and molecular vibration, respectively. The values of $E_{GM}\left( h \right)$ were obtained from simulation results shown in **Figure S2a**. The frequency of $E_{GM}$ under VSC was determined by exponential curve fitting of the $E_{GM}$ versus *h* data. The CHO fit showed excellent agreement with the simulated data as shown in **Figure 3b**, yielding $g$ = 0.007 eV (corresponding to ℏΩ_R_ = 113 cm^-1^). From the eigenvectors $\alpha$ and $\beta$, the Hopfield coefficients $\alpha_{UP,LP}^{2}$ and $\beta_{UP,LP}^{2}$ were calculated to quantify the contribution of each polaritonic mode to the grating and molecular vibrations, respectively:

$\alpha_{UP,LP}^{2}=\frac{g^{2}}{\left( E_{GM}-E_{UP,LP} \right)^{2}+g^{2}}$ (S10)

$\beta_{UP,LP}^{2}=1-\alpha_{UP,LP}^{2}$ (S11)

When a PMMA slab was placed on top of the graphene-integrated deep Ag grating, the coupling involved the grating mode ($E_{GM}$), the molecular vibration ($E_{m}$ at 1730 cm^-1^), and two GP modes ($E_{GP1}$ at 1778 cm^-1^ and $E_{GP2}$ at 1678 cm^-1^) (**Figure 4**). Consequently, the CHO model was extended to a 4 x 4 matrix Hamiltonian to capture the coupling among these four resonances [^[[5]](#endnote-5)^]:

$\left( \begin{matrix} E_{\text{GM}}\left( h \right) & g_{1} & g_{2} & g_{3} \\ g_{1} & E_{\text{GP1}} & 0 & 0 \\ g_{2} & 0 & E_{m} & 0 \\ g_{3} & 0 & 0 & E_{\text{GP2}} \end{matrix} \right)\left( \begin{matrix} \alpha_{1} \\ \alpha_{2} \\ \begin{matrix} \alpha_{3} \\ \alpha_{4} \end{matrix} \end{matrix} \right)=E\left( \begin{matrix} \alpha_{1} \\ \alpha_{2} \\ \begin{matrix} \alpha_{3} \\ \alpha_{4} \end{matrix} \end{matrix} \right)$ (S12)

Here, $g_{1}$, $g_{2}$, and $g_{3}$ denote the coupling strengths between $E_{GM}\left( h \right)$ and $E_{GP1}$, $E_{GM}\left( h \right)$ and $E_{m}$, and $E_{GM}\left( h \right)$ and $E_{GP2}$. Solving equation (S12) yields four energy eigenstates, which are plotted as black dashed lines in **Figure 4b**. The CHO fitting produced coupling strengths of $g_{1}$ = 0.003 eV, $g_{2}$ = 0.005 eV, and $g_{3}$ = 0.003 eV. Hopfield coefficients were obtained from the eigenvectors by employing the ‘eig’ function in MATLAB.

The strong coupling was evaluated by comparing the Rabi splitting (Ω) to the average linewidths of the oscillators involved. The linewidths were determined as follows: the grating mode had a linewidth (𝛤_g_) of 134 cm^-1^, the bare PMMA layer (𝛤_PMMA_) had 25 cm^-1^, and graphene on a deep Ag grating (𝛤_graphene_) had 14 cm^-1^. The corresponding absorption spectra are shown in **Figure S2b**-**d**. Since our system involves four oscillators (the grating, PMMA, and two graphene plasmon modes), the average linewidth is calculated to be 46.75 cm^-1^. Rabi splitting values at *h* = 0.97 μm, obtained from **Figure 4b**, are Ω_1_ = 35.63 cm^-1^, Ω_2_ = 60.81 cm^-1^, and Ω_3_ = 36.12 cm^-1^. Of these, only Ω_2_ exceeds the average linewidth. Therefore, only coupling associated with Ω_2_ meets the criterion for strong coupling, whereas Ω_1_ and Ω_3_ remains in the weak coupling regime.

**Dispersion relation and propagation length of graphene plasmons.**

The dispersion relation of graphene plasmons is given by [^[[6]](#endnote-6)^]

$q\left( \omega\right)={2i\varepsilon_{0}\bar{\varepsilon} \omega}/{\sigma\left( \omega\right)}$ (S13)

where, $\varepsilon_{0}$ is the permittivity of free space, $\bar{\varepsilon}$ is the average dielectric constant between air and the substrate, and $\sigma\left( \omega\right)$is the complex conductivity of graphene. The propagation length of the plasmons ($L_{P}$) is expressed as [^[[7]](#endnote-7)^]

$L_{P}=1/q^{"}$ (S14)

where $q^{"}$ is the imaginary part of the plasmon wavevector.

Therefore, when the trench gap is filled with PMMA, $\bar{\varepsilon}$ increases due to the high dielectric constant of PMMA. This results in an increase in plasmon loss and a decrease in the $L_{P}$, which suppresses the intensity of the graphene plasmon resonance in the PMMA-filled deep Ag grating.


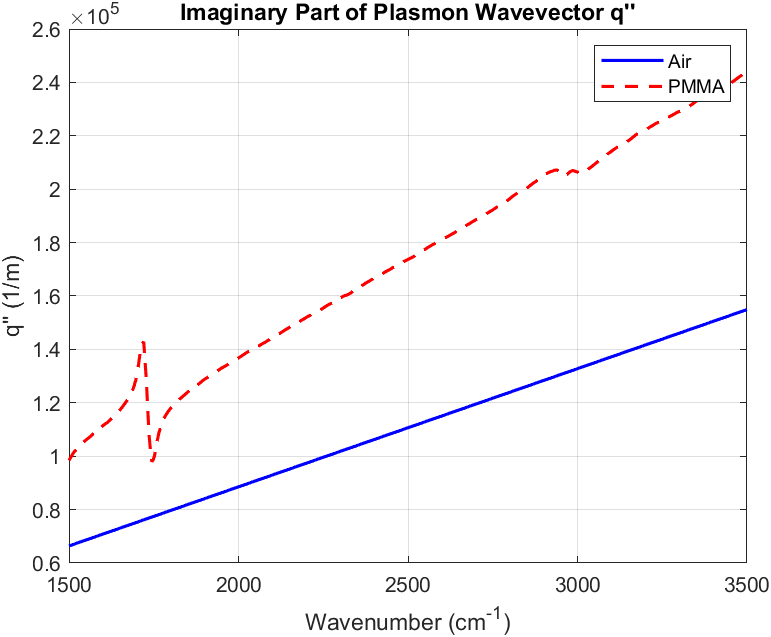

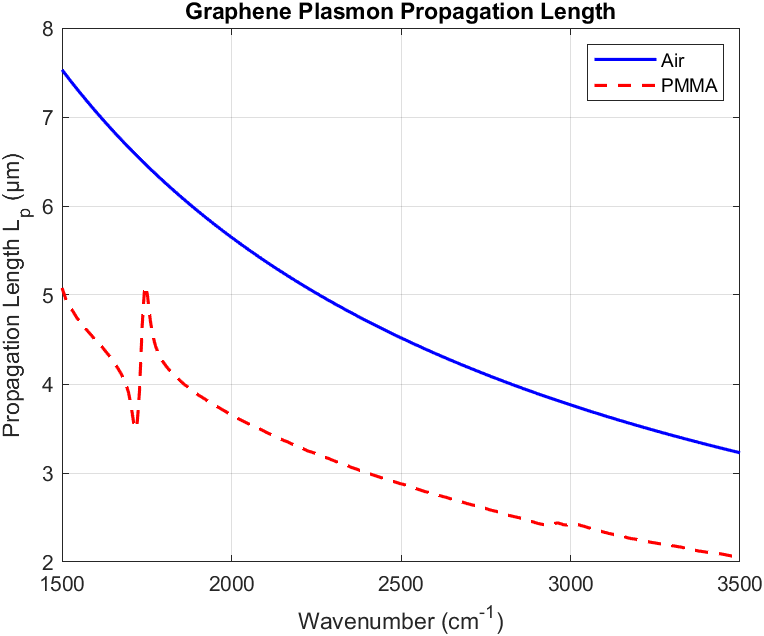


The imaginary part of the GP dispersion ($q^{"}$) and the propagation length of plasmons ($L_{P}$) for the deep Ag grating with its gap filled with air (blue solid line) and with PMMA (red dashed line).


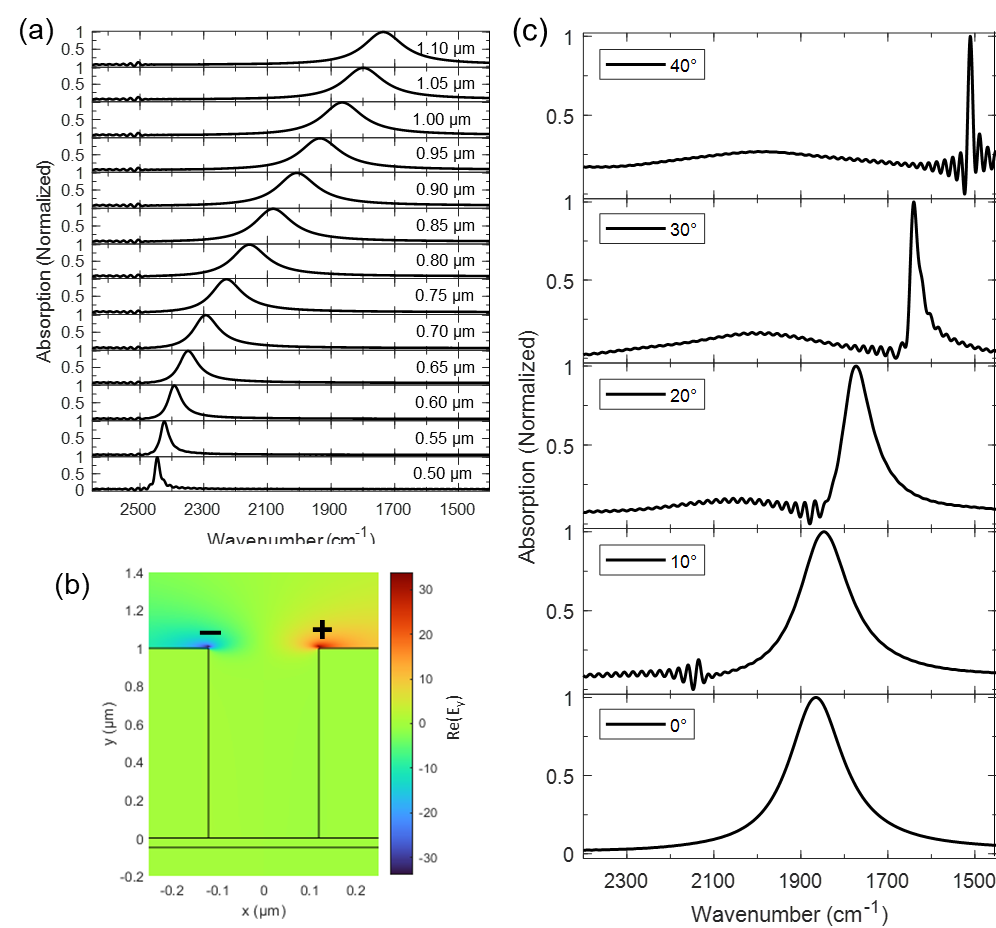


**Figure S1.** A bare deep Ag grating structure. (a) Absorption spectra of the bare deep Ag grating structure (Λ = 4 μm, *b* = 240 nm, and *t* = 50 nm) with the grating height, *h*, varied from 0.5 to 1.1 μm. (b) The real part of the *y*-component of the *E*-field intensity map, Re(*E_y_*), showing antisymmetric *E*-field distributions of the SP modes at the edges of a trench opening, denoted by + and - signs (*h* = 1.0 μm). (c) Angle-dependent absorption spectra of the deep Ag grating (*h* = 1.0 μm) with the incident angle of light varied from 0 to 40°, indicating the formation of the SP modes.


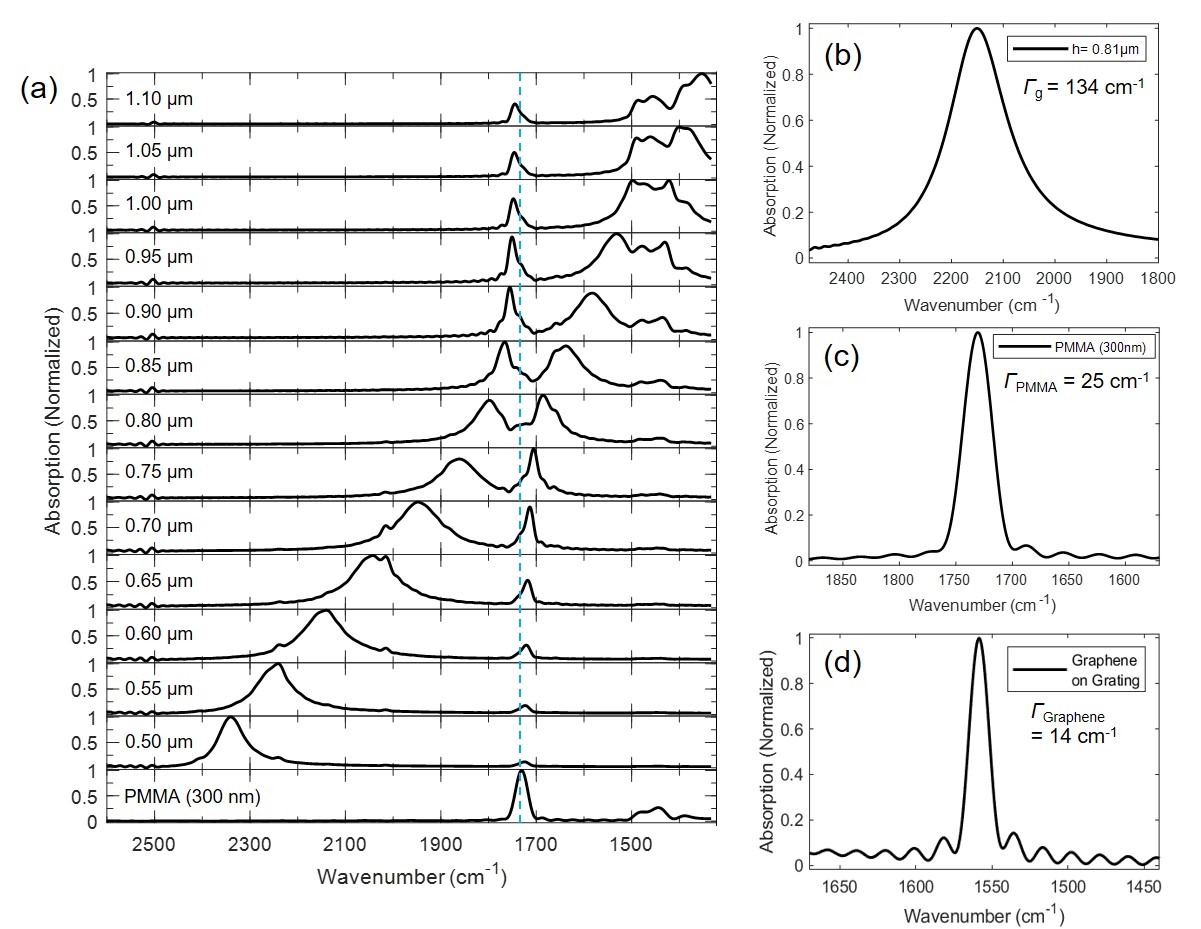


**Figure S2.** Absorption spectra of a graphene-integrated deep Ag grating with PMMA filled in the trench gap. (a) Absorption spectra with *h* varied from 0.5 to 1.1 μm. The absorption spectrum of a 300 nm-thick PMMA layer is shown at the bottom of the figure, with the C=O stretching mode of PMMA indicated by a blue dotted line. (b) Absorption spectrum of a deep metal grating (Λ = 4 μm, *b* = 240 nm, *t* = 50 nm, and *h* = 0.81 μm) showing the full-width at half-maximum, 𝛤_g_, of 134 cm^-1^. (c) Absorption spectrum of a bare PMMA layer showing 𝛤_PMMA_ of 25 cm^-1^. (d) Absorption spectrum of graphene on a deep Ag grating showing 𝛤_graphene_ of 14 cm^-1^.


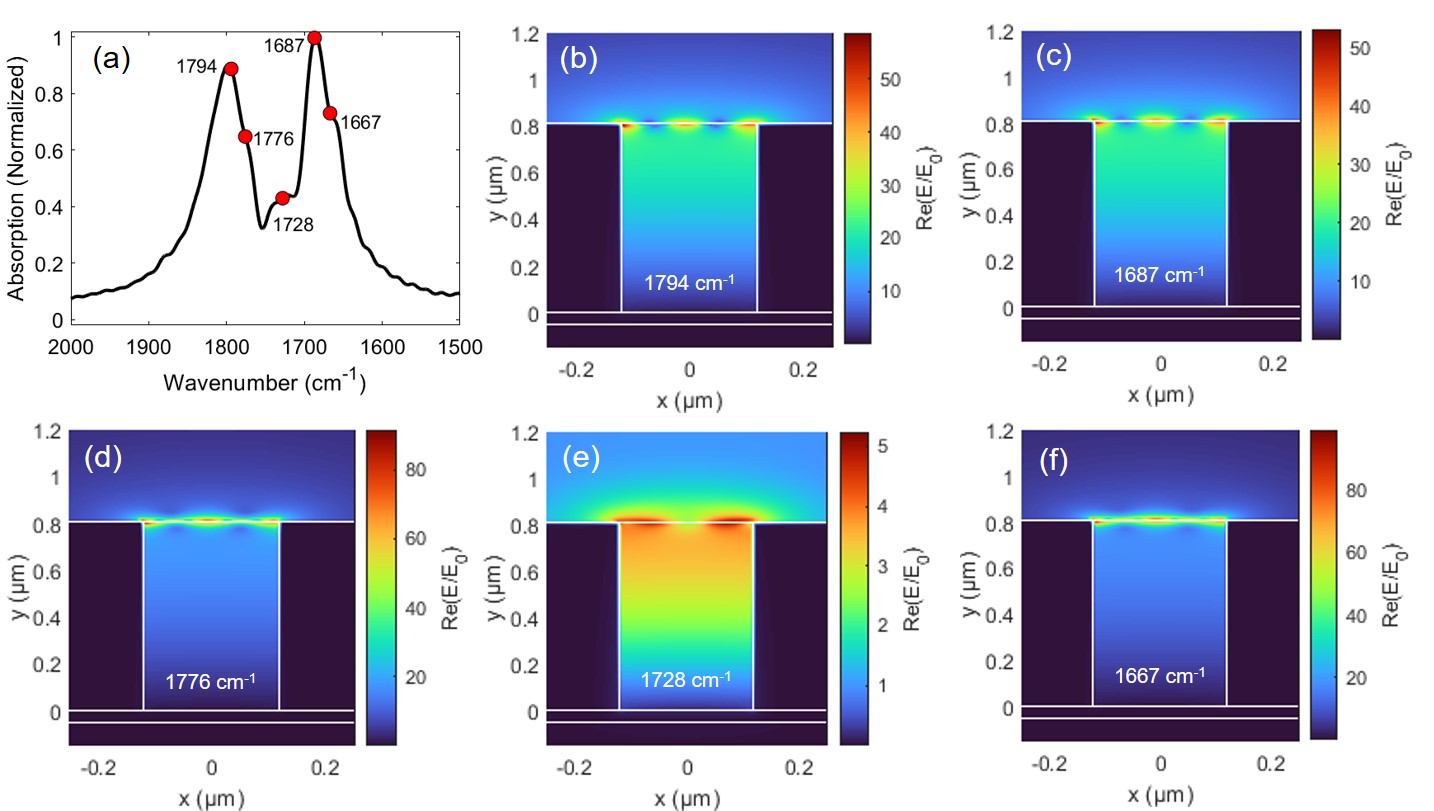


**Figure S3.** *E*-field intensity maps of a graphene-integrated deep Ag grating with PMMA filled in the trench gap. The *E*-field intensity maps were simulated at the frequencies indicated in the absorption spectrum (a) corresponding to the UP (b, 1794 cm^-1^), LP (c, 1687 cm^-1^), GP1 mode (d, 1776 cm^-1^), the C=O stretching mode of PMMA (e, 1728 cm^-1^), and GP2 mode (f, 1667 cm^-1^).


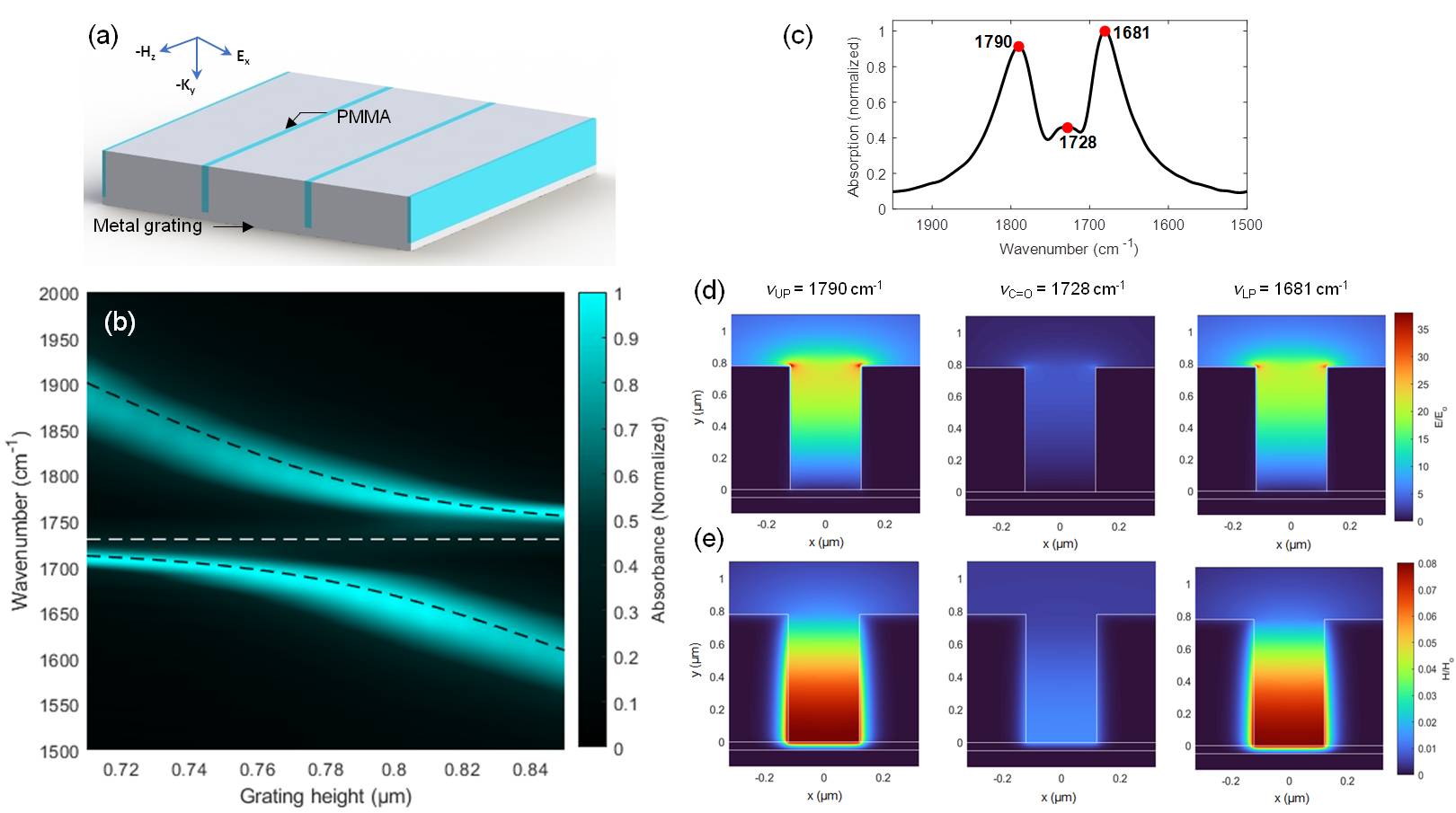


**Figure S4.** Graphene-free deep Ag grating with PMMA filled in the grating gap. (a) Schematic diagram. (b) Absorption intensity map with the *h* values varied from 0.71 to 0.85 μm. A CHO model fit (black dashed lines) suggests a ℏΩ_R_ of 111 cm^-1^. A white dashed line indicates the frequency of the C=O stretching mode of PMMA. (c) Absorption spectrum at *h* = 0.78 μm. (d) *E*- and (e) *H*-field intensity maps at the frequencies of the UP (1790 cm^-1^), the C=O stretching mode of PMMA (1728 cm^-1^), and LP mode (1681 cm^-1^).


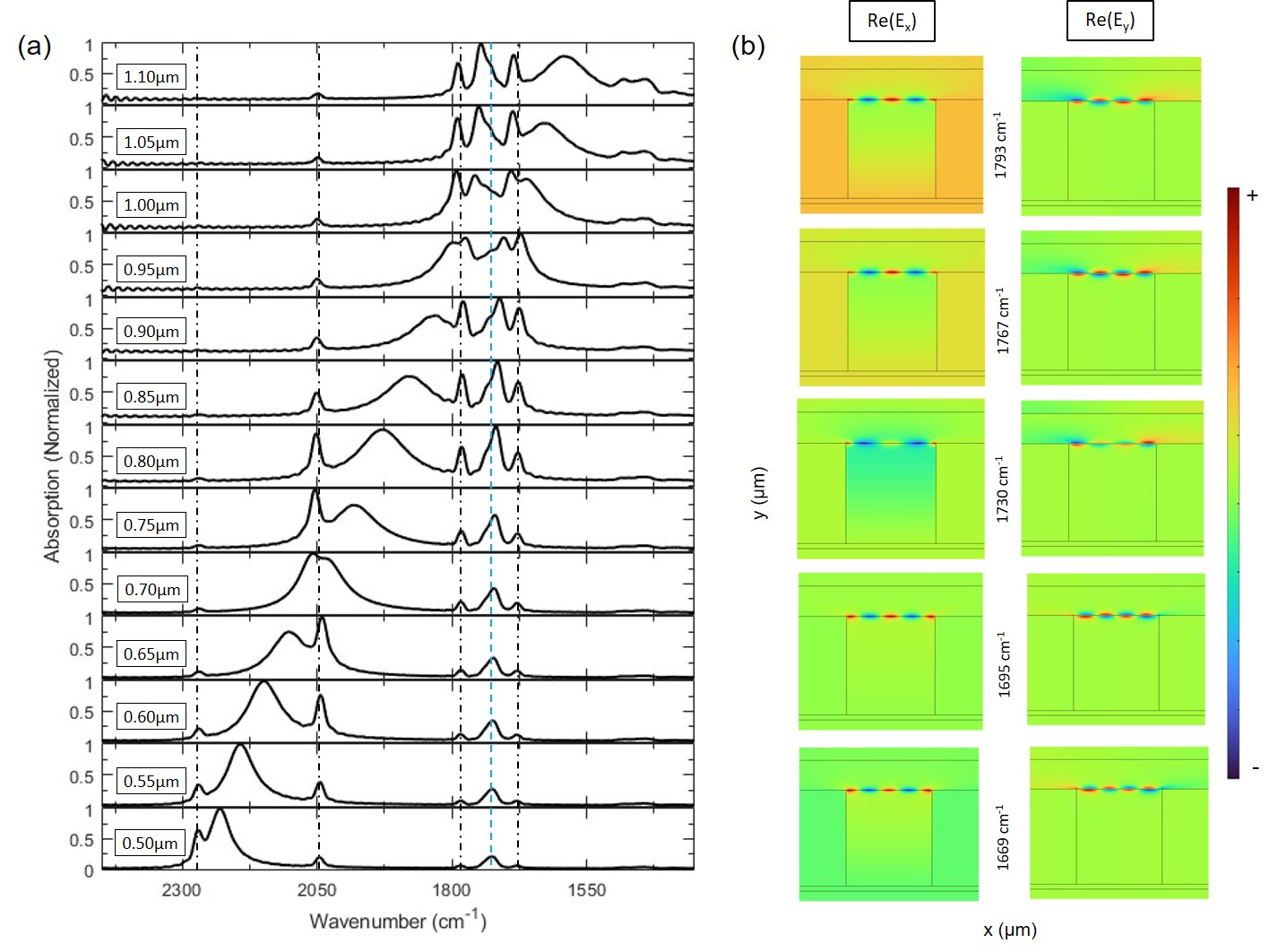


**Figure S5.** Graphene-integrated deep Ag grating with a PMMA layer on top of graphene. (a) Absorption spectra with *h* varied from 0.5 to 1.1 μm (Λ = 4 µm, *b* = 0.24 µm, PMMA thickness = 300 nm and *µ* = 0.5 eV). Black dash-dot lines indicate the frequencies of GP modes, while the blue dashed line marks the C=O stretching mode of PMMA. (b) Real *E*-field component intensity maps for the *x*- (*E_x_*) and *y*-direction (*E_y_*) at the frequencies of the UP (1793 cm^-1^), MUP (1767 cm^-1^), MLP (1695 cm^-1^), LP (1669 cm^-1^), and the C=O stretching mode of PMMA (1730 cm^-1^) at *h* = 0.97 μm. The deep Ag grating supports strong *E*-field in both x- and y-direction, as shown by the *E_x_* and *E_y_* maps, which exhibit pronounced GP modes at all UP, MUP, MLP, and LP frequencies. Notably, the graphene-free deep Ag grating supports *E*-fields only at the trench openings and exclusively in the y-direction, as shown in the Re(*E_y_*) map in **Figure S1b**.


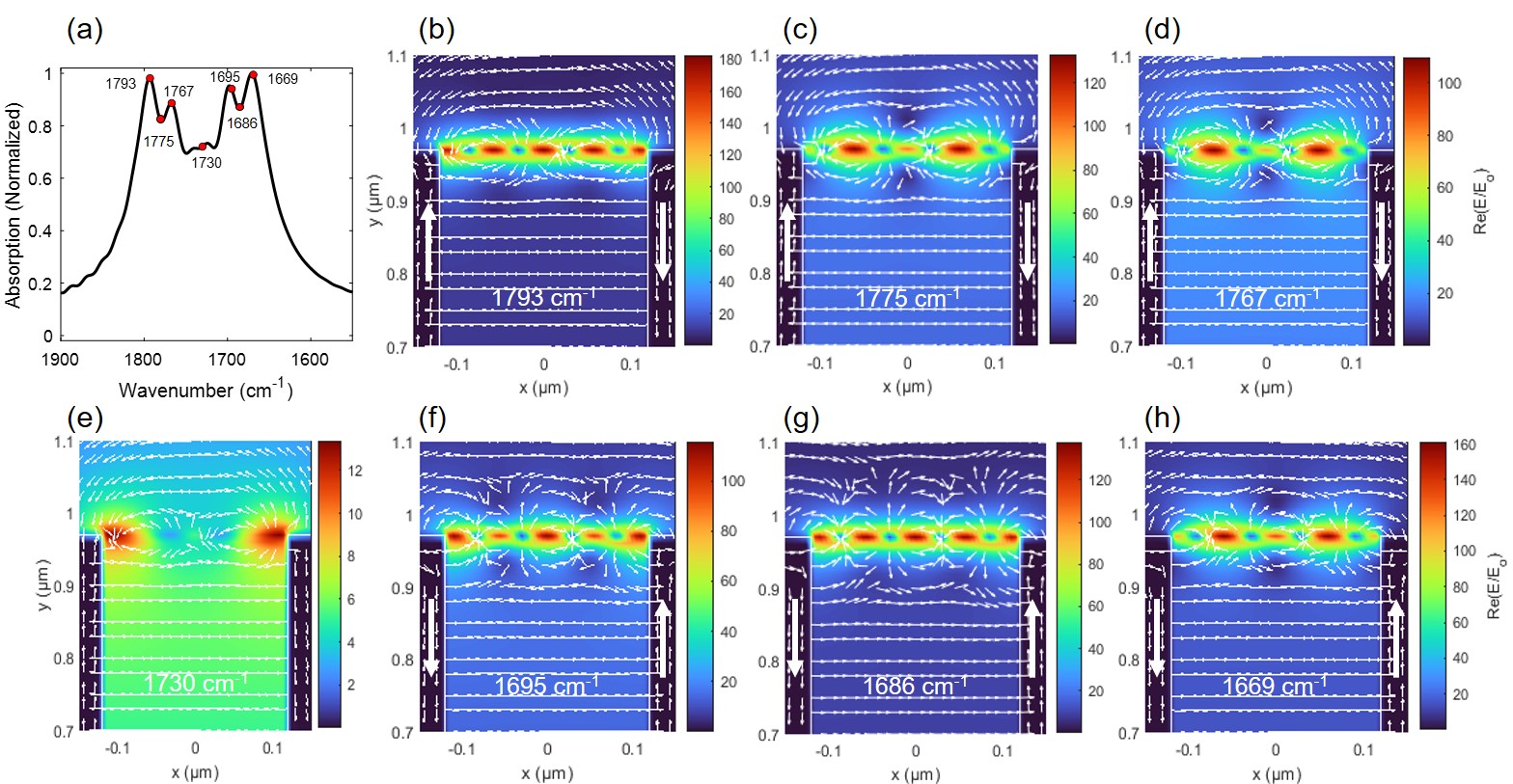


**Figure S6.** *E*-field intensity maps of the graphene-integrated deep Ag grating with a PMMA layer on top of graphene. (a) Absorption spectrum for grating parameters: Λ = 4 µm, *b* = 0.24 µm, *h* = 0.97 μm, PMMA thickness = 300 nm, and *µ* = 0.5 eV. *E*-field intensity maps were simulated at the peak frequencies of the UP (b, 1793 cm^-1^), MUP (d, 1767 cm^-1^), MLP (f, 1695 cm^-1^), and LP (h, 1669 cm^-1^); frequencies of two GP modes (c, 1775 cm^-1^ and g, 1686 cm^-1^); and the frequency of the PMMA C=O stretching mode (e, 1730 cm^-1^). At all frequencies of UP, MUP, MLP, LP, and GP modes, the *E*-field is concentrated along the graphene layer. Strong GP modes manifest as nodes and antinodes in the field intensity, causing the electric vector fields to circulate at the interface between PMMA, graphene, and the air gap within the deep Ag grating. In contrast, the GP mode disappears at the PMMA C=O stretching frequency, resulting in *E*-field intensity localized primarily at the edges of the trench openings in (e).

*
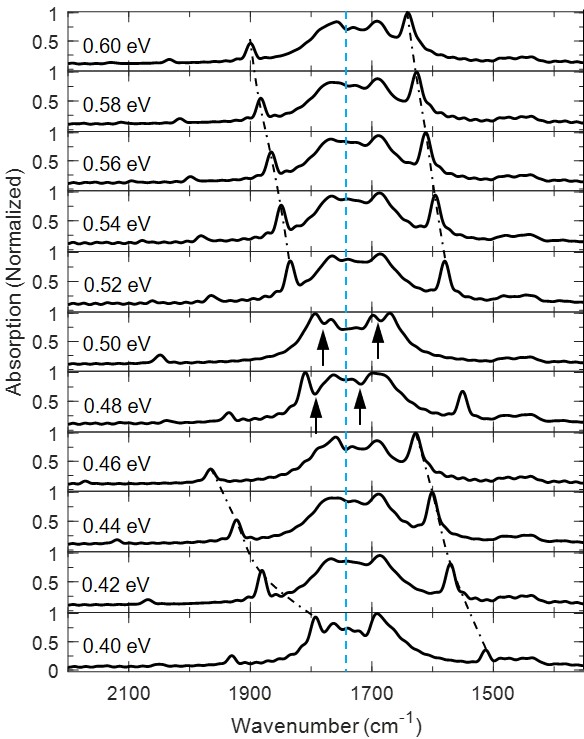
*

**Figure S7.** Tunable VSC by varying the chemical potential applied to graphene. Absorption spectra were shown with the chemical potential *μ* varied from 0.40 to 0.60 eV in increments of 0.02 eV. The frequency of the GP mode was continuously tuned and coupled to the polaritonic states formed between the grating modes and molecular vibrations. Black arrows at *μ* = 0.48 and 0.50 eV highlight the abrupt shifts of the GP mode frequencies toward the polaritonic states, causing the spectral dips in the grating mode. A blue dashed line marks the frequency of the PMMA C=O stretching mode at 1730 cm^-1^. Dashed-dot lines show the GP modes shifting linearly when the GP modes are off-resonant from the polaritonic states (0.40 - 0.46 eV and 0.52 - 0.60 eV).


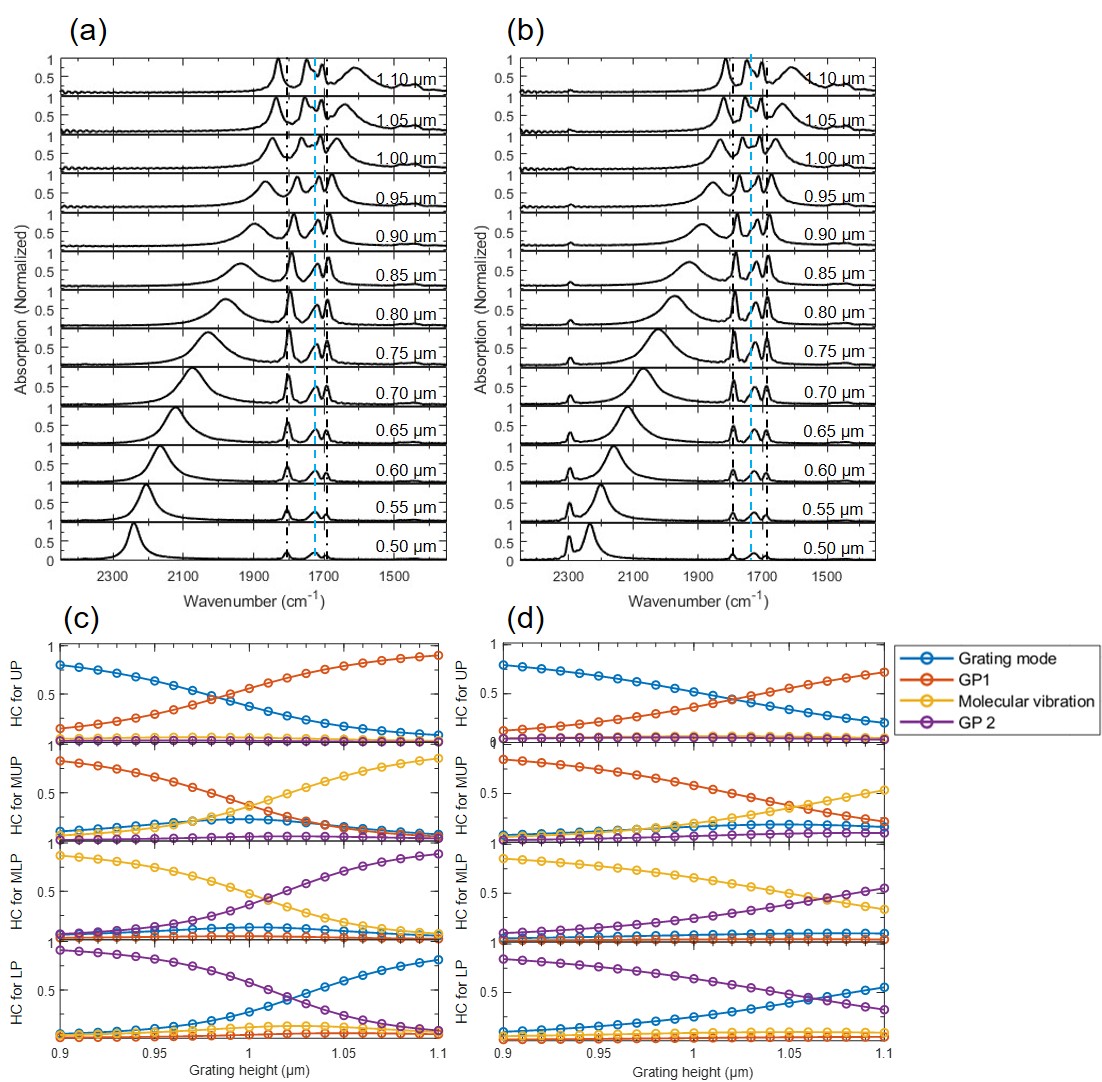


**Figure S8.** Tunable VSC by varying the number of graphene layers. Absorption spectra of the deep Ag grating integrated with (a) double-layer and (b) triple-layer graphene, with the *h* varied from 0.50 to 1.10 μm. A blue dashed line indicates the frequency of the PMMA C=O stretching mode, and dashed-dot lines indicate the frequencies of the GP modes. Hopfield coefficients for the UP, MUP, MLP, and LP show mode mixing between the grating mode (blue), GP1 (orange), molecular vibration (yellow), and GP2 mode (purple) for the grating with (c) double-layer and (d) triple-layer graphene.

**References**

1. [] B. Zhao and Z. M. Zhang, “Study of magnetic polaritons in deep gratings for thermal emission control,” *Journal of Quantitative Spectroscopy & Radiative Transfer*, vol. 135, pp. 81-89, 2014, https://doi.org/10.1016/j.jqsrt.2013.11.016. [↑](#endnote-ref-1)
2. [] H. F. Ghaemi, T. Thio, D. E. Grupp, T. W. Ebbesen, and H. J. Lezec, “Surface plasmons enhance optical transmission through subwavelength holes,” *Phys. Rev. B.*, vol. 58, no. 11, pp. 6779-6782, 1998, https://doi.org/10.1103/PhysRevB.58.6779. [↑](#endnote-ref-2)
3. [] V. Savona, L. C. Andreani, P. Schwendimann, and A. Quattropani, “Quantum well excitons in semiconductor microcavities: unified treatment of weak and strong coupling regimes,” *Solid State Communications*, vol. 93, no. 9, pp. 733-739, 1995, https://doi.org/10.1016/0038-1098(94)00865-5. [↑](#endnote-ref-3)
4. [] M. S. Skolnick, T. A. Fisher, and D. M. Whittaker, “Strong coupling phenomena in quantum microcavity structures,” *Semiconductor Science and Technology*, vol. 13, no. 7, pp. 645-669, 1998, DOI: 10.1088/0268-1242/13/7/003. [↑](#endnote-ref-4)
5. [] M. Son, Z. T. Armstrong, R. T. Allen, A. Dhavamani, M. S. Arnold, and M. T. Zanni, “Energy cascades in donor-acceptor exciton-polaritons observed by ultrafast two-dimensional white-light spectroscopy,” *Nature Communications*, vol. 13, pp. 7305, 2022, https://doi.org/10.1038/s41467-022-35046-2. [↑](#endnote-ref-5)
6. [] M. Jablan, H. Buljan, and M. Soljačić, ”Plasmonics in graphene at infrared frequencies,” *Physical Review B*, vol. 80, no. 24, pp. 245435, 2009, https://doi.org/10.1103/PhysRevB.80.245435. [↑](#endnote-ref-6)
7. [] F. Karimi, A. H. Davoody, and I. Knezevic, “Dielectric function and plasmons in graphene: A self-consistent-field calculation within a Markovian master equation formalism,” *Physical Review B*, vol. 93, no. 20, pp. 205421, 2016, https://doi.org/10.1103/PhysRevB.93.205421. [↑](#endnote-ref-7)
